# DNPTrapper: an assembly editing tool for finishing and analysis of complex repeat regions

**DOI:** 10.1186/1471-2105-7-155

**Published:** 2006-03-20

**Authors:** Erik Arner, Martti T Tammi, Anh-Nhi Tran, Ellen Kindlund, Bjorn Andersson

**Affiliations:** 1 Karolinska Institute, Center for Genomics and Bioinformatics, Stockholm, Sweden

## Abstract

**Background:**

Many genome projects are left unfinished due to complex, repeated regions. Finishing is the most time consuming step in sequencing and current finishing tools are not designed with particular attention to the repeat problem.

**Results:**

We have developed DNPTrapper, a shotgun sequence finishing tool, specifically designed to address the problems posed by the presence of repeated regions in the target sequence. The program detects and visualizes single base differences between nearly identical repeat copies, and offers the overview and flexibility needed to rapidly resolve complex regions within a working session. The use of a database allows large amounts of data to be stored and handled, and allows viewing of mammalian size genomes. The program is available under an Open Source license.

**Conclusion:**

With DNPTrapper, it is possible to separate repeated regions that previously were considered impossible to resolve, and finishing tasks that previously took days or weeks can be resolved within hours or even minutes.

## Background

High-throughput methods for genome sequencing, in combination with increased computer power and better algorithms for sequence assembly, have yielded a plethora of genomes accessible for analysis. However, complicated parts of sequenced genomes tend to be left unfinished to a large extent. This is especially the case for eukaryotic genomes, where the majority of the genomes presently sequenced have repeated regions that were left unresolved (see e.g. [1] and [2] for discussion on how duplications affect eukaryotic genome projects). Current shotgun sequencing assembly programs are not designed to handle long stretches of repeated DNA in the target sequence, and it is common that repeated sequences are left out of the assembly altogether. In addition, repeats often cause assembly errors, e.g. large artificial rearrangements due to misassembled repeat regions. Also common are assemblies with the repeat copies merged into alignments of high coverage, with reads of the repeat region piled on top of each other. Although many repeats appear to have no discernible biological function, in many cases the repeats play an important role in the biology of the organism [3], and some organisms have a significant amount of their genes organized into head-to-tail tandem arrays consisting of nearly identical genes. One example is *Trypanosoma cruzi*, a protozoan parasite with a highly repetitive genome [4] containing multi-copy gene families such as cruzipain [5], histone H1 [6] and HSP70 [7].

The presence of repeated regions in the target sequence is thus the key problem in shotgun sequencing. This is especially true for the whole genome shotgun (WGS) approach that has emerged as the method of choice in recent years. Where the previous clone-by-clone strategies allowed for compartmentalizing the genome and handling of repeat regions locally, the WGS approach requires handling of all copies of a repeat region simultaneously, even if they are spread throughout the genome. The problems caused by repeats can be somewhat reduced by combining the two approaches, but the incidence of repeats remains a key problem and major cause of errors in shotgun sequencing assemblies.

A successful strategy for solving the problem for short repeat regions has been the use of mate pairs [8]. Using mate pairs it is possible to correctly assemble tandem repeat regions or single repeat units dispersed in unique genomic sequence, depending on the order in which the fragments are assembled and providing that a sufficient amount of the sequence reads sampling the repeat copies have mate pairs in the unique regions. However, this strategy fails when nearly identical repeats are organized in tandem stretches longer than twice the shotgun fragment insert length. In this case, the mate pairs of reads sampling repeat units, sample another part of the same repeat region, which makes it impossible for current assembly algorithms to determine the correct layout of the shotgun fragment reads.

These problems of the common assembly methods place a heavy burden on the biologists working on the finishing stage of sequencing projects and add to the bottleneck that finishing constitutes. A number of tools have been developed to aid this process [9-12]. However, these tools, although very useful for non-repeated sequences, are not designed for finishing complex, repeated regions. Generally, a major problem with current finishing tools is that they provide either a close-up view of the shotgun reads in the different contigs of the assembly, or a zoomed out view of the entire genome, with nothing in between. With a rigid close-up view, the user can only view a small portion of the repeat region at a time, and much scrolling is required in order to get a clear understanding of the region, whereas a genome wide view does not allow for manual inspection and correction at the read level. Furthermore, common tools generally lack the flexibility needed to correct obvious errors in a straight forward fashion, often requiring the user to re-run the whole assembly and hope for the reads to end up in the correct positions. Most importantly, although other systems use high quality mismatches between reads in attempt to separate repeats, none of them have adequate specificity and fully utilize the presence of single base differences between repeat copies as a resource in repeat resolution.

We here present DNPTrapper, an assembly editing and visualization tool specifically designed for manual analysis and finishing of repeated regions. It differs from previous tools by providing flexibility and an overview that greatly simplifies the finishing process, by allowing the user to view whole repeat regions at once and to edit assembly errors manually by drag and drop. The program implements and visualizes the results of a previously described statistical method that detects defined nucleotide positions (DNPs, representing single base differences between repeat units) in the presence of sequencing errors [13].

The source code is available from the authors under an Open Source license.

## Implementation

### Overview

DNPTrapper is a sequence alignment editing tool developed for finishing complicated shotgun sequencing projects. It combines relatively simple but powerful algorithms with visualization of problematic assembly locations, thus providing detailed information for biologists and allowing rapid decision making to make necessary corrections. The main goal of DNPTrapper is to provide more power to the user than other finishing tools. The user can move sequences using drag and drop; re-align them; cut, copy, and paste them; run algorithms on them; add and remove features; choose between view modes; zoom in and out. The user interface is a front end to a database, and changes made using Trapper are automatically propagated to the database. DNPTrapper is essentially an editor that visualizes assemblies of shotgun sequence fragment reads as gapped multiple alignments. The assemblies can be produced by any assembler that produces the supported file formats (e.g. .ace-files from Phrap), and can be exported to the same format after repeat analysis and resolution. In addition to the read sequences, different features and data such as DNPs, vector sequence, quality values, chromatograms and mate pairs are visualized in the editor according to the preferences of the user. Sequence features can be present in the input assembly files, or be added during the finishing process by running default built-in algorithms that detect and label the desired features.

**Figure 1:**
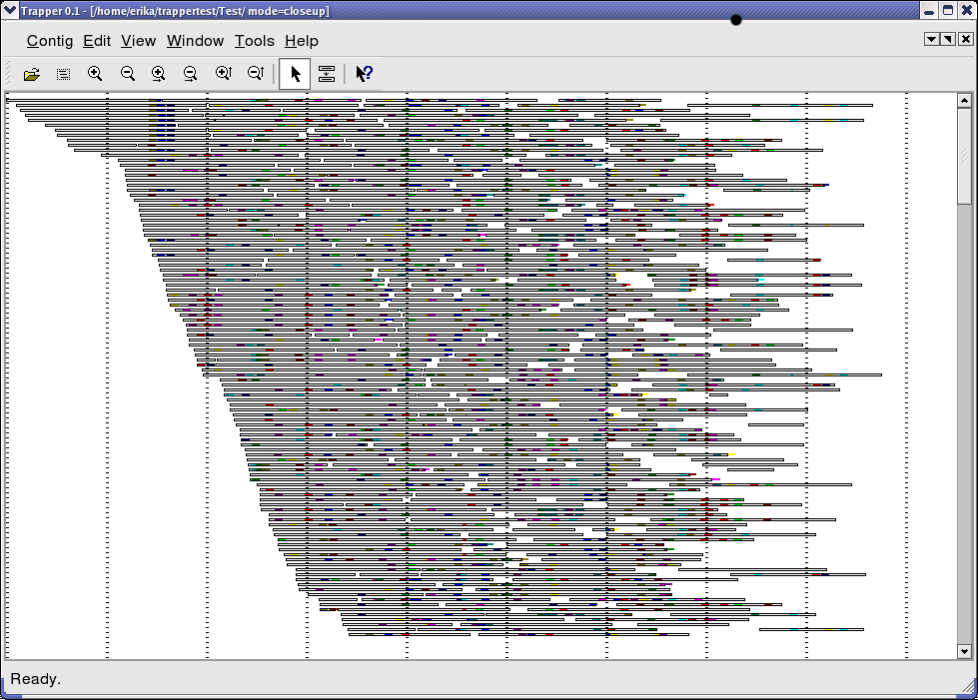
Partial view of an alignment in DNPTrapper. Boxes represent shotgun fragment reads, colored dots represent DNPs. This is a zoomed out view of a simulated dataset after DNP detection. The difference between repeat copies was 2% between any two units. At this zoom level, DNP patterns are visible.

Finishing of repeated regions is made less complicated using DNPTrapper by providing three key features that common finishing tools lack. The first feature is the availability of a birds-eye view of all the reads in a contig. By zooming out, the user gets an overview of the contig, its length and depth, and can get a clear picture of the general properties of the region at once. In combination with the second key feature, the built-in DNP visualization, this is very powerful for resolving repeats that differ in only a few positions along the repeat unit. The DNPs are color coded, which makes it possible to distinguish different repeat groups in a high coverage alignment by eye (Figure 1). Whereas other finishing tools can be quite rigid in the editing choices available to the user, DNPTrapper also allows for manual editing operations such as cut, copy and paste. In this respect, DNPTrapper is similar to editors from other problem domains, e.g. word processing software. This flexibility offered to the user is the third key feature of the program. It enables the user to correct errors in the assembly directly in the program window, without having to re-run the previous assembly step, and also to use a "sand-box" approach, which allows a user to try out different editing solutions in parallel within a single session. By using drag and drop, it is possible to sort reads into different repeat groups according to DNP content. By default, only vertical movement of reads is allowed to avoid breaking the overall layout of the assembly but horizontal moving of reads followed by re-aligning can also be performed.

Together with default built-in algorithms for sorting reads according to a variety of criteria (e.g. DNP content), these features allow for semi-automatic resolution of almost identical repeats in a straight forward fashion, reducing finishing time for such regions from days to a matter of hours or even minutes. Below, the flow of a typical session with DNPTrapper is outlined, with description of some key aspects of program use.

#### Importing data

The first step in a DNPTrapper session is to import data into the editor. File formats currently supported are the Phrap .ace format and a native XML format described in the documentation. Other formats will be added; in the meantime there are free converters between different file formats available at the AMOS website [14]. After importing, contigs in the project are available for analysis. In the default visualization mode after opening a contig, sequence reads are represented by black-bordered boxes on white background. When zooming in, the base sequences and quality values, visualized by a grey-scale, become visible. The user can customize the visualization mode of quality values and other features.

#### ReAligning

Since most assembly programs produce assemblies that are locally non-optimal, the next step is to apply the built-in ReAlign algorithm [15] to the contigs that appear to contain repeats. This step is crucial for the subsequent application of the DNP algorithm.

#### DNP method

When the selected contig has been ReAligned, the DNP algorithm can be run with the desired parameters (see [13] for details) in order to detect single base differences between repeat copies. These appear as color coded dots in the alignment. The different colors represent different DNP types, where a type is defined by the consensus base and the base of the single base difference. There are thus twelve different DNP types and twelve corresponding colors.

#### Repeat resolution

**Figure 2:**
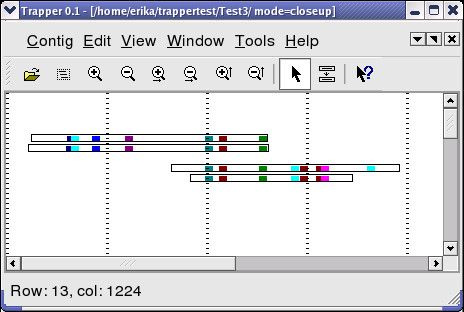
DNP walking. Manual repeat resolution is carried out by starting with a read and locating other reads with the same DNP pattern. The repeat group is extended to the right and left by locating reads with the same DNPs at the ends of the reads. In this simple example, the two top reads constitute the beginning of a repeat group. The group is extended by identification of the same DNP pattern in the left end of the two bottom reads. This can be done by eye or searching the alignment for reads with the same DNP ID.

When the DNPs have been detected, the actual finishing begins. Using the DNPs the reads can be separated into different repeat groups, and the color coding of the DNPs makes it possible to distinguish patterns in the alignment visually. Two different techniques are available in order to perform the separation. One is to start with a read, select one of its DNPs and automatically locate and group all other reads containing a DNP of the same type at the same column in the alignment. Another DNP present in the group can subsequently be selected to locate other reads that belong in the same group. In this way, the user "walks" along the DNPs in a group to extend it in both directions until all the DNPs of the group have been exhausted (Figure 2). The same procedure is subsequently performed on the reads that are left after extraction of the group. Individual reads or groups of reads can be dragged in and out of repeat groups as the user sees fit.

Another strategy is to start by applying a sorting algorithm to the contig before finishing. It sorts the reads into different groups according to their DNP content by picking out a read and locating all other reads sharing DNPs with the original read and adding them to the set. Since the newly found reads may contain additional DNPs, the process is repeated until all the DNPs in the set have been exhausted. However, the DNP detection method may produce false positives, and it is often necessary to perform manual correction of the group assignments after sorting since groups may have been merged together due to erroneous DNP assignments. Still, this simple algorithm performs remarkably well due to the low error rate of the DNP detection method, and errors are easy to resolve using human judgment and other available data such as mate pairs and chromatograms. The most straightforward way to use DNPTrapper is to use the sorting procedure and resolve the remaining ambiguities manually.

#### Exporting data

After analysis and resolution, the database can be exported as a flat file (currently available formats are .ace and the native XML format), so that the data can re-enter the normal finishing pipeline. There are also other export options that are more suitable if the objective is analysis rather than finishing; arbitrary subsets of sequences, or their consensus sequence, can be extracted and written to file in FASTA format for analysis with other tools.

### Implementation

DNPTrapper has been implemented using C++ and relies on Open Source Software. The graphical user interface library is Qt [16], and the Berkeley Database [17] is used for storing the data.

Great care has been taken to make the system suitable for projects of varying sizes. All data is stored in the database and is read from a disc on request from the GUI. This makes the RAM requirement low, allowing handling of projects of virtually any size. This includes, but is not limited to, mammalian size genomes. The parsers used for importing data into DNPTrapper are event-based and file size is thus not a limiting factor.

Apart from the scalability, flexibility has also been a major design goal. A plug-in system and a well documented Application Program Interface makes adding new algorithms, visualization modes and sequence features simple.

DNPTrapper currently runs under Linux Fedora Core 3 and have been tested on 32 and 64 bit platforms. Ports to other platforms may be carried out in the future.

## Results

In order to further illustrate the functionality of DNPTrapper, we have tested it on three data sets, one simulated and two from the *T. cruzi *whole genome shotgun sequencing project [4]. In all three cases, the reads from each project were assembled with Phrap and subsequently imported into DNPTrapper for further analysis. Phred quality values were not used in the assembly steps, in order to make sure that all reads with sequence similarity ended up aligned to each other. The use of quality values causes Phrap to partially resolve repeated regions, which makes for en incomplete analysis. Instead, the parameter -default_qual was set to 10. Regions of 89% average Phred quality or more in the reads was subjected to DNP analysis.

The simulated data set was included as a proof of concept, to verify the correctness of the implementation of the DNP method and the functionality of the program. The regions from *T. cruzi *were chosen since they are examples of complicated genomic regions of biological importance (both contain genes), and regions where the assembly program (Celera assembler, [18]) has failed to assemble the reads correctly. Furthermore, they constitute two different types of tandem repeats that can be resolved using DNPTrapper. These genes have not been characterized previously in *T. cruzi*. The regions were located by scanning the assembly for regions with unusually high shotgun coverage, and reads matching these regions where extracted by performing sequence similarity searches in the read database.

### Simulated project

We simulated shotgun sequencing of a 20 kb template sequence consisting of 10 repeat units of 2 kb in tandem, with a pair-wise sequence difference between any two repeat copies of 2%. The simulation was performed using *sim_gun *(described in [19]), an in-house shotgun sequencing simulation program that emulates the sequencing process as closely as possible. It uses quality values from real shotgun projects when constructing reads at random places in the target sequence.

**Figure 3:**
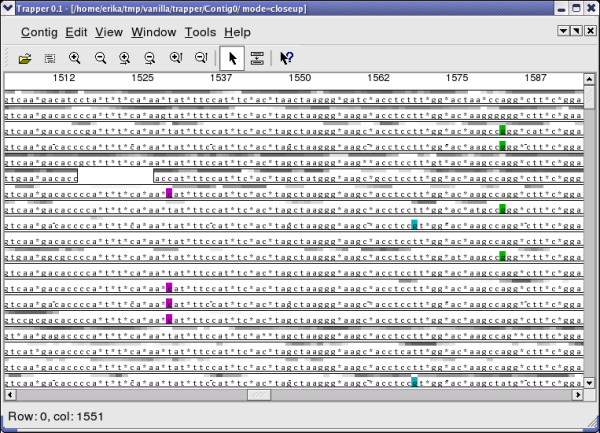
Zoomed in view of the simulated dataset. The different colors of the DNPs represent different DNP types. For instance, the light green DNPs indicate that the single base difference is an 'a' while the consensus base is a 'g'. This color coding makes it easy to observe DNP patterns in the alignment. Gaps are denoted by '*' in the alignment.

Figure 1 shows a zoomed out view of the data after ReAligning and DNP detection. The colored dots represent the detected DNPs. Figure 3 shows the same alignment, but at a closer zoom level. This level is often the only available one in other finishing tools (e.g. Consed).

**Figure 4:**
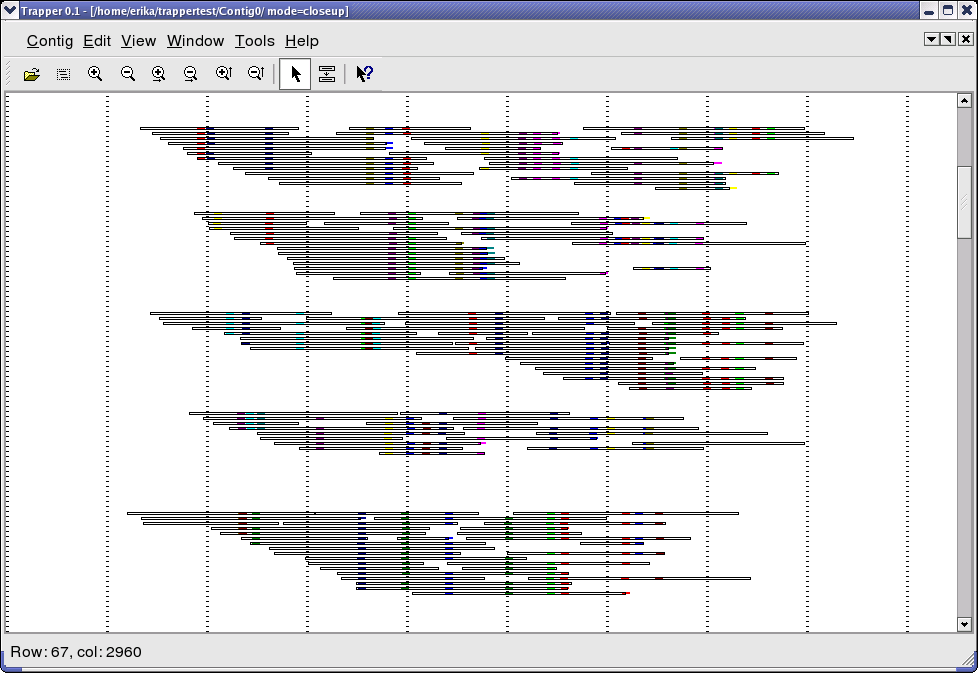
Five of the ten repeat groups in the simulated dataset after automatic sorting and manual correction. Each group consists of reads sampled from one repeat unit in the target sequence respectively. The repeat groups are clearly distinguishable from each other by their DNP patterns.

**Figure 5:**
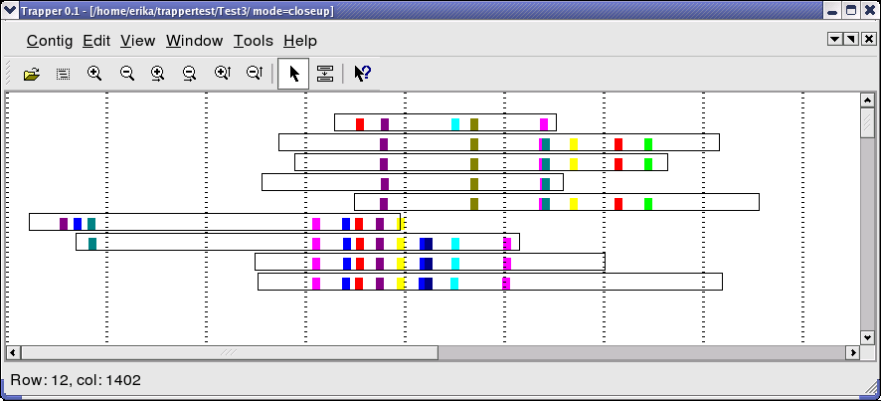
Zoomed in view of an erroneous merging of two repeat groups by the DNP sorting algorithm. The error is caused by two sequencing errors in the read at the top, where two bases (first red and third cyan) have been erroneously sequenced into DNP bases of another group.

This dataset could be resolved and divided into the ten repeat groups known to be present within minutes using the DNP sorting algorithm built into DNPTrapper in combination with manual curation (Figure 4). The algorithm performed the division almost single-handedly, apart from a case where erroneous DNP assignments had caused two groups to merge (Figure 5). This error was easily corrected manually by moving reads directly in the DNPTrapper window.

### Elongation factor 2 (EF2)

The consensus sequence of the *T. cruzi *whole genome shotgun assembly contains a region with strong sequence homology to EF2, crucial for the translocation step in eukaryote protein synthesis [20]. The assembly program had assembled 202 shotgun reads covering this region into one pile, unable to separate the repeat copies. The sequence similarity search located an additional 140 reads from this gene that had not been included in the original assembly.

**Figure 6:**
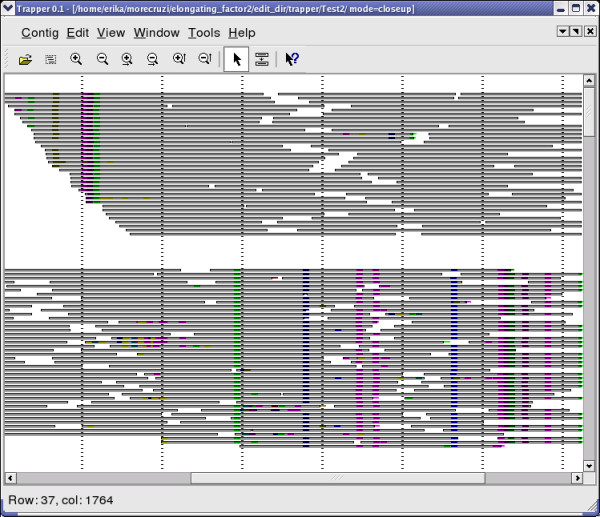
EF2 assembly after division into two groups according to DNP content. Mate pair analysis of the two groups confirmed the two groups are located on different homologs.

The Phrap assembly of these 342 reads was readily divided into two large groups (Figure 6) using a combination of manual and automatic sorting by DNP content. Within each group, no further division was possible. The region illustrates a previously observed phenomenon in *T. cruzi*, where genes repeated in tandem are conserved within the repeat arrays on each allelic chromosome locus and more divergent between homologs. In this analysis, the division into two groups was confirmed by examining the distribution of mate pairs in the assembly (mated read distance 2–3 kb and 3–4 kb). No reads in one group had mate pairs in the other group, which supports the hypothesis that this is indeed a case where the repeats are virtually identical within one homolog and differ across homologs. The mechanism behind this phenomenon is currently unknown; this separation of the two haplotypes, pinpointing their differences, enables further studies.

### Monoglyceride lipase (MGL)

Another region present in the *T. cruzi *assembly is homologous to MGL, which is part of the fat digestion pathway [21]. Again, the reads sampling this region had been assembled in one pile consisting of 71 reads. Another 439 reads matching this region, discarded by the assembly program, were also located in the read database.

This repeat region was found to have a different structure than the EF2 region described above. Instead of two large groups corresponding to conserved repeat arrays on each homolog, the reads could be divided into several small groups representing repeat units present in one or two copies each.

**Figure 7:**
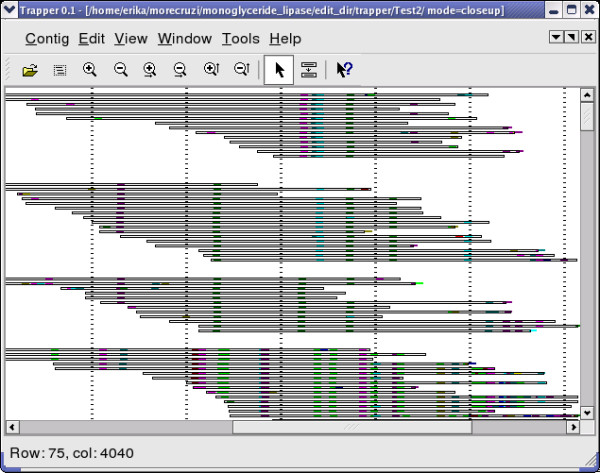
Four of the 25 different repeat groups found after manual repeat resolution. A coverage analysis of the different groups indicate that the majority of the repeat units are present in copy numbers of one or two, with an additional three units with copy number three to eight.

The Phrap alignment consisted of 510 reads and had a maximum coverage of 285 reads. Due to the fact that a high coverage yields more erroneous DNP assignments, most of the repeat separation had to be performed manually since false DNP positives have a bad effect on the DNP sorting algorithm. Still, within one hour's work by one person, 25 distinct repeat groups could be identified and separated (Figure 7), with their respective unique sites pinpointed. A coverage analysis of the different groups indicated that four repeat units were present in one copy, seven were present in two copies, and eleven were present in one or two copies. Three additional groups had higher coverage, indicating copy numbers between three and eight. Again, the reason for this arrangement in the parasite genome is not understood, much due to the fact that such regions previously have been too difficult to resolve and characterize. With the single base differences between repeat copies identified, it becomes possible to study such regions in detail and determine the functional consequences of the different variants present in repeat arrays.

## Discussion

Repeated regions remain the key problem in shotgun sequencing. Current assembly algorithms are unable to assemble nearly identical repeats correctly. Repeated parts of the target genome are routinely left out of the assembly altogether, and when they are included, the resulting assemblies are full of errors such as large rearrangements, merged repeat copies and broken scaffolds.

Finishing is the major bottleneck in sequencing projects, and complex, repeated regions are often left unresolved. Despite the fact that repeats are the main reason for assembly errors, commonly used finishing tools, e.g. Consed, are not designed with the repeat problem in mind. The software is very useful for gap closure and other finishing operations on non-repeated sequences. However, when repeats are encountered, current tools lack in flexibility and overview, not allowing the user to correct obvious errors manually and presenting a rigid, too close-up view of the repeat region. Moreover, no current finishing software utilizes single base differences between repeat copies as a tool for repeat separation.

With this in mind, we have developed a shotgun sequencing assembly editor specifically designed to cope with the problems encountered when the target sequence contains nearly identical repeats. By zooming out of a contig, the user gets an overview of its length and depth, and the color coding of DNPs makes it possible to directly see patterns of repeat groups in the data, which allows for rapid manual repeat separation. Instead of only allowing assembly programs to determine the positions of reads, the user is allowed to drag and drop sequences into the positions they see fit. The option of re-assembly is still available, and the number of options for finishing is therefore increased. These three features – DNP method, overview, and flexibility – set this tool apart from other finishing software and introduce a new concept for finishing.

The structure of DNPTrapper makes it possible to add new features. Several possible extensions can already be envisaged. Most important is the addition of an algorithm that uses mate pairs to order the resolved repeat groups. Other improvements include supporting more input file formats, adding more viewable features, and adding a global view of all contigs in a project, with specific algorithms for comparing, ordering and merging contigs. Also, work is in progress to interface DNPTrapper to TIGR's open source assembler AMOS [14].

## Conclusion

Our results show that finishing tasks previously deemed impossible to resolve or very time consuming can be performed in a straight-forward fashion using DNPTrapper. Using this tool, the process of resolving repeat regions that would take days or weeks using current software, instead can be resolved within hours or even minutes. The use of DNPTrapper as a finishing tool reduces finishing times and thus costs. It also allows in-depth studies and characterization of the poorly understood repeat regions present in most genomes.

## Availability and requirements

**Project name: **DNPTrapper

**Project home page: **

**Operating system: **Linux

**Programming language: **C++

**License: **BSD Open Source license

**Any restrictions to use by non-academics: **No restrictions

## Authors' contributions

EA, MTT and BA planned the project. EA implemented the algorithms and supervised implementation of the GUI. EK and ANT acquired the data. All authors read and approved the final manuscript.

## References

[B1] She X, Jiang Z, Clark RA, Liu G, Cheng Z, Tuzun E, Church DM, Sutton G, Halpern AL, Eichler EE (2004). Shotgun sequence assembly and recent segmental duplications within the human genome. Nature.

[B2] Eichler EE, Clark RA, She X (2004). An assessment of the sequence gaps: unfinished business in a finished human genome. Nat Rev Genet.

[B3] Ji Y, Eichler EE, Schwartz S, Nicholls RD (2000). Structure of chromosomal duplicons and their role in mediating human genomic disorders. Genome Res.

[B4] El-Sayed NM, Myler PJ, Bartholomeu DC, Nilsson D, Aggarwal DG, Tran AN, Ghedin E, Worthey EA, Delcher AL, Blandin G, Westenberger SJ, Caler E, Cerqueira GC, Branche C, Haas B, Anupama A, Arner E, Åslund L, Attipoe P, Bontempi E, Bringaud F, Burton P, Cadag E, Campbell DA, Carrington M, Crabtree J, Darban H, da Silveira JF, de Jong P, Edwards K, Englund PT, Fazelina G, Feldblyum T, Ferella M, Frasch AC, Gull K, Horn D, Hou L, Huang Y, Kindlund E, Klingbeil M, Kluge S, Koo H, Lacerda D, Levin MJ, Lorenzi H, Louie T, Machado CR, McCulloch R, McKenna A, Mizuno Y, Mottram JC, Nelson S, Ochaya S, Osoegawa K, Pai G, Parsons M, Pentony M, Pettersson U, Pop M, Ramirez JL, Rinta J, Robertson L, Salzberg SL, Sanchez DO, Seyler A, Sharma R, Shetty J, Simpson AJ, Sisk E, Tammi MT, Tarleton R, Teixeira S, Van Aken S, Vogt C, Ward PN, Wickstead B, Wortman J, White O, Fraser CM, Stuart KD, Andersson B (2005). The genome sequence of *Trypanosoma cruzi*, etiologic agent of Chagas disease. Science.

[B5] Campetella O, Henriksson J, Aslund L, Frasch AC, Pettersson U, Cazzulo JJ (1992). The major cysteine proteinase (cruzipain) from Trypanosoma cruzi is encoded by multiple polymorphic tandemly organized genes located on different chromosomes. Mol Biochem Parasitol.

[B6] Aslund L, Carlsson L, Henriksson J, Rydaker M, Toro GC, Galanti N, Pettersson U (1994). A gene family encoding heterogeneous histone H1 proteins in Trypanosoma cruzi. Mol Biochem Parasitol.

[B7] Requena JM, Lopez MC, Jimenez-Ruiz A, de la Torre JC, Alonso C (1988). A head-to-tail tandem organization of hsp70 genes in Trypanosoma cruzi. Nucleic Acids Res.

[B8] Edwards A, Caskey CT (1990). Closure strategies for random DNA sequencing methods. A Companion to Methods in Enzymology.

[B9] Gordon D, Abajian C, Green P (1998). Consed: A graphical tool for sequence finishing. Genome Res.

[B10] Staden R, Beal KF, Bonfield JK (2000). The Staden Package, 1998. Methods Mol Biol.

[B11] Gordon D, Desmarais C, Green P (2001). Automated finishing with Autofinish. Genome Res.

[B12] Frangeul L, Glaser P, Rusniok C, Buchrieser C, Duchaud E, Dehoux P, Kunst F (2004). CAAT-Box, contigs-Assembly and Annotation Tool-Box for genome sequencing projects. Bioinformatics.

[B13] Tammi MT, Arner E, Britton T, Andersson B (2002). Separation of nearly identical repeats is shotgun assemblies using defined nucleotide positions, DNPs. Bioinformatics.

[B14] AMOS home page. http://www.tigr.org/software/AMOS/.

[B15] Anson EL, Myers EW (1997). Realigner: a program for refining DNA sequence multi-alignments. J Comp Biol.

[B16] QT home page. http://www.trolltech.com.

[B17] Berkeley DB home page. http://www.sleepycat.com.

[B18] Myers EW, Sutton GG, Delcher AL, Dew IM, Fasulo DP, Flanigan MJ, Kravitz SA, Mobarry CM, Reinert KH, Remington KA, Anson EL, Bolanos RA, Chou HH, Jordan CM, Halpern AL, Lonardi S, Beasley EM, Brandon RC, Chen L, Dunn PJ, Lai Z, Liang Y, Nusskern DR, Zhan M, Zhang Q, Zheng X, Rubin GM, Adams MD, Venter JC (2000). A whole-genome assembly of Drosophila. Science.

[B19] Tammi MT, Arner E, Andersson B (2003). TRAP: Tandem Repeat Assembly Program produces improved shotgun assemblies of repetitive sequences. Comput Methods Programs Biomed.

[B20] Arlinghaus R, Shaeffer J, Schweet R (1964). Mechanism of peptide bond formation in polypeptide synthesis. Proc Natl Acad Sci USA.

[B21] Senior JR, Isselbacher KJ (1963). Demonstration of an intestinal monoglyceride lipase: an enzyme with a possible role in the intracellular completion of fat digestion. J Clin Invest.

